# Identification and mapping of QTLs and their corresponding candidate genes controlling high night‐time temperature stress tolerance in wheat (*Triticum aestivum* L.)

**DOI:** 10.1002/tpg2.20517

**Published:** 2024-09-24

**Authors:** Kaviraj S. Kahlon, Kanwardeep S. Rawale, Sachin Kumar, Kulvinder S. Gill

**Affiliations:** ^1^ Department of Crop and Soil Sciences Washington State University Pullman Washington USA; ^2^ Geneshifters LLC Pullman Washington USA; ^3^ Department of Botany/Department of Genetics and Plant Breeding Chaudhary Charan Singh University Meerut Uttar Pradesh India

## Abstract

With every 1°C rise in temperature, yields are predicted to decrease by 5%–6% for both cool and warm season crops, threatening food production, which should double by 2050 to meet the global demand. While high night‐time temperature (HNT) stress is expected to increase due to climate change, limited information is available on the genetic control of the trait, especially in wheat (*Triticum aestivum* L.). To identify genes controlling the HNT trait, we evaluated a doubled haploid (DH) population developed from a cross between an HNT tolerant line KSG1203 and KSG0057, a selection out of a mega variety PBW343 from South East Asia that turned out to be HNT susceptible. The population, along with the parents, were evaluated under 30°C night‐time (HNT stress) keeping the daytime temperature to normal 22°C. The same daytime and 16°C night‐time temperature were used as a control. The HNT treatment negatively impacted all agronomic traits under evaluation, with a percentage reduction of 0.5%–35% for the tolerant parent, 8%–75% for the susceptible parent, and 8%–50% for the DH population. Performed using sequencing‐based genotyping, quantitative trait locus (QTL) mapping identified 19 QTLs on 13 wheat chromosomes explaining 9.72%–28.81% of cumulative phenotypic variance for HNT stress tolerance, along with 13 that were for traits under normal growing conditions. The size of QTL intervals ranged between 0.021 and 97.48 Mb, with the number of genes ranging between 2 and 867. A candidate gene analysis for the smallest six QTL intervals identified eight putative candidates for night‐time heat stress tolerance.

## Core Ideas

- 19 Quantitative trait loci (QTLs) controlling high night‐time temperature (HNT) stress tolerance in wheat were localized to 13 regions of 21 kb to 97.48 Mb size.
- QTLs for HNT stress vary from daytime heat stress in the same population, indicating unique tolerance mechanisms.
- The smallest six regions were less than 3.5 Mb in size and contained 2–27 genes per region.
- Using comparative genomics with gene expression, eight candidate genes for four agronomic traits were identified.
- The smallest QTL interval was 21.3 kb for tiller number, which carried a known heat stress tolerance KH‐I domain.

**Abbreviations**
BM: biomass
DH: doubled haploid
DTH: days to heading
GY: grain yield
HNT: high night‐time temperature
PH: plant height
PVE: percent variance explained
QTL: quantitative trait locus
SN: spikelet number per spike
SNP: single nucleotide polymorphism
TN: tiller number
TSW: total spike weight

## 1 INTRODUCTION

Crop production should double by 2050 to meet the global demand of a rapidly growing population, which faces many challenges including biotic and abiotic stresses. As per recent estimates, increase in global night‐time temperature due to climate change will be 1.4 times higher than that for the daytime temperature (IPCC, 2023). Bread wheat (*Triticum aestivum* L.) is one of the major cereal crops, annually grown on ∼216 million ha globally and accounts for 20% of global calories and protein intake (Erenstein et al., 2022). Being a cool‐season crop, wheat is extremely sensitive to temperature increases and fluctuations during various plant developmental stages (Cossani & Reynolds, 2012). Every 1°C rise in temperature above the threshold has been shown to result in 5%–6% yield loss in wheat (Tack et al., 2015). These estimates are for the day‐time heat stress. The corresponding estimates for the high night‐time temperature (HNT) stress are, however, not known. In this study, we show that even the most successful wheat varieties in the world are highly sensitive to HNT, and, thus, are vulnerable to the climate change impact.

Physiological processes underpinning plant development are differentially regulated by the day and night‐time temperatures. In Arabidopsis (*Arabidopsis thaliana*), transcriptome analysis revealed that ∼75% of the heat‐responsive transcripts show a time‐of‐day dependent response (Swindell et al., 2007), suggesting that genes controlling night‐heat stress tolerance are different from that controlling daytime heat stress. While several studies have been conducted to observe the effect of day‐time heat stress on plant development, genetic, molecular, and physiological controls of HNT stress tolerance, especially in crop plants, are largely unknown. Meta‐analysis across various plant functional types revealed a negative effect of HNT stress on plant yield through biomass (BM) allocation in reproductive organs (P. Jing et al., 2016). Additionally, a negative effect of HNT stress was observed on photosynthesis in rice (*Oryza sativa* L.) and wheat (Narayanan et al., 2015; Peraudeau et al., 2015). In addition to plant yield, HNT stress was shown to negatively impact plant BM and stem elongation in tomatoes (*Solanum lycopersicum*) (Ohtaka et al., 2020). Genes or quantitative trait loci (QTLs) controlling HNT stress tolerance have not been reported in any of the plants.

In wheat, some reports have studied effects of daytime heat stress on genetic, metabolic, and physiological processes. A meta‐analysis revealed 441 QTLs for 31 heat‐responsive traits in wheat with eight major QTL clusters on chromosomes 1B, 2B, 2D, 4A, 4B, 4D, 5A, and 7A controlling drought and heat‐stress tolerance (Acuña‐Galindo et al., 2015; Kumar, Singh, et al., 2021). However, no information is available about the effect of HNT stress on the development and performance of the wheat plant and no genes/QTLs have so far been identified.

The present study was designed to study the impact of HNT stress in wheat on various agronomic traits, and identify QTLs, chromosomal regions, and the underlying genes controlling the HNT stress tolerance. As the same population was also used to identify QTLs and genes controlling daytime heat stress (S. Kumar et al., 2024), we have also performed comparison of the genetic control of the two types of heat stresses.

## 2 MATERIALS AND METHODS

### 2.1 Doubled haploid mapping population

A global collection of ∼1200 wheat accessions was made from the area around the equator that was expected to carry heat stress tolerance while reducing the redundancy among the selected lines (Amita Mohan and Kulvinder Gill, unpublished data). The collection was screened using five different controlled condition heat screening protocols, including one for HNT stress tolerance, along with field evaluation of the selected lines for 2 years at six locations in India. Composite data analyses of the controlled condition and field‐based screening resulted in the selection of seven heat tolerant lines, including KSG1203, containing complementary mechanisms of heat stress tolerance (Climate Resilient Wheat Innovation Lab, team led by Kulvinder Gill; https://agrilinks.org/activities/climate‐resilient‐wheat‐innovation‐lab).

Evaluation of the collection revealed that most of the collected accessions were mixtures; thus, single plant selections were made from each line and were given different names. The KSG1203 line was a selection out of an Egyptian variety Giza168. Therefore, we recommend that seeds of KSG1203 should be obtained from Washington State University rather than using Giza168 for further studies.

To simultaneously identify QTLs and genes controlling HNT stress tolerance and to transfer it into popular varieties of South East Asia, a doubled haploid (DH) mapping population of 136 plants was developed from a cross between a heat‐tolerant parent line KSG1203 and a mega variety, KSG0057 (a selection out of PBW343), that ruled wheat production in South East Asia for more than 10 years. The wheat–maize DH method (Laurie & Reymondie, 1991) with modifications was used to develop the DH population. Briefly, fresh maize (*Zea mays* L.) pollen was used to fertilize wheat spikes 2 days after emasculation. On day 3, 213 mg/L of 2,4‐d with a pH 10.36 was sprayed on spikes. After 16 days from pollination, embryos were regenerated using ½ MS medium in glass tubes and chromosome doubling was done via 0.05% colchicine treatment. Seeds of the individual DH plants were multiplied under controlled conditions (22°C day for 16 h and 18°C night). Spikes of the individual plants were covered using emasculation bags to avoid cross‐pollination. Pure seeds of the DH plants were used for the study, along with that of the two parents.

### 2.2 Phenotypic evaluation of the DH population under HNT stress

Two adjoining greenhouses at the Wheat Growth Facility at Washington State University with accurately controlled temperature and light conditions were used for the study, one for the control condition evaluation and the other for HNT stress treatment. The greenhouses have a central air conditioning system with air passing through high‐efficiency particulate air (HEPA) filters and temperature variation between or within greenhouses is minimal. Sixteen plants for each DH line and 48 plants for each parental line were individually planted in 4 × 12 in. (1.6 L) tall pots (MT49, mini‐treepots) filled with Sunshine Mix LC1 (Sun Gro Horticulture Distribution Inc.). At the two‐leaf stage, plants for each DH and parental line were randomly divided into two groups, one for the experiment and the second for the control. Multiple plants of each DH line were randomly distributed through the greenhouse to account for any bench‐to‐bench variation. Plant growing conditions in the control greenhouse were set at 22°C Day for 16 h (6 a.m. to 10 p.m.) and 16°C night. Conditions in the HNT stress treatment greenhouse were set at the same conditions as that for control experiment except for the night‐time temperature that was set at 30°C. Starting at 10 p.m., the night‐time temperature was gradually increased to 30°C by 11 p.m. The 30°C temperature was maintained for 5 h before ramping it down to 22°C by 6 a.m. All other conditions including watering, fertilizer, pest control, and lights were kept the same between the two greenhouses. Both greenhouses were supplemented with evenly spaced 400 W sodium halide lamps to maintain a light intensity of 300 W/m^2^ during the day.

**Core Ideas**

- 19 Quantitative trait loci (QTLs) controlling high night‐time temperature (HNT) stress tolerance in wheat were localized to 13 regions of 21 kb to 97.48 Mb size.
- QTLs for HNT stress vary from daytime heat stress in the same population, indicating unique tolerance mechanisms.
- The smallest six regions were less than 3.5 Mb in size and contained 2–27 genes per region.
- Using comparative genomics with gene expression, eight candidate genes for four agronomic traits were identified.
- The smallest QTL interval was 21.3 kb for tiller number, which carried a known heat stress tolerance KH‐I domain.

### 2.3 Phenotypic data collection

The phenotypic data were collected on seven agronomic traits that were suspected to be affected by HNT stress: (1) days to heading (DTH), (2) SN, (3) plant height (PH), (4) tiller number (TN), (5) total aboveground BM, (6) total spike weight (TSW), and (7) grain yield (GY) per plant. Except for DTH, data for various phenotypic traits were collected at maturity. DTH was recorded as DTH (Zadoks scale 53) when quarter of the main spike emerged out of the flag leaf (Zadoks et al., 1974). The TN was recorded by counting the spike‐bearing tillers at maturity. For each trait, the effect of HNT stress was recorded by taking percentage change relative to the control, using the following formula: {(C − HNT)/C} × 100.

### 2.4 Sequencing‐based genotyping (SBG) and data analysis

Genotyping of the DH population was performed by Geneshifters, LLC (info@geneshifters.com) via their SBG procedure, which is designed to enrich sequencing for the genic fraction of the genome and is also explained elsewhere (S. Kumar et al., 2024). Briefly, the genomic DNA of the DH population and the two parents was extracted using the SDS (sodium dodecyl sulfate)‐based DNA isolation protocol (Randhawa et al., 2009). Genomic DNA was normalized to a concentration of ∼100 ng/µL before shipping the samples for SBG. After re‐normalizing DNA concentration to 10 ng/µL, sequencing library preparation, enrichment for genic fraction, quality check, and sequencing were performed. Average ∼1 million reads of average 120 bp length each were generated for each DH line, whereas for each of the two parents, 5 million reads were generated.

For sequence data analysis, raw sequencing data were demultiplexed using a barcode splitter program (Hannon, 2010). Individual raw reads of the parental genotypes were aligned to the IWGSC high‐confidence wheat gene annotation version 2 using the *bowtie2* mapping program at the default settings (https://www.wheatgenome.org/) (Langmead & Salzberg, 2012; The International Wheat Genome Sequencing Consortium [IWGSC] et al., 2018). For each sample, duplicate reads were removed and a consensus sequence for the mapped reads was developed using the *CAP3* program (Huang, 1999). The de novo consensus sequence of the parental genotypes was used to map reads of the DH population using the *bowtie2* (Langmead & Salzberg, 2012) mapping program. Single nucleotide polymorphism (SNP) calling was done using the *Vcftools* program and SNPs with more than 80% allele call and a minimum sequencing depth of 10 reads were retained for further analysis (Danecek et al., 2011). SNP call data were sorted based on the wheat physical reference genome and were converted to “A” or “B” format using the standalone *tassel* package (Bradbury et al., 2007).

### 2.5 Statistical analysis and physical QTL mapping

The mean for various phenotypic traits was calculated in Microsoft Excel (Microsoft Corporation, 2018). Statistical analysis of the phenotypic data was performed using the *GraphPad* software (https://www.graphpad.com/). The mean of different phenotypic traits and “AB” formatted genotyping data of the DH population sorted using the physical coordinates of the wheat reference genome were used for QTL mapping. Composite interval QTL mapping was performed using the R/QTL package and a permutation test with 1000 iterations and QTLs with an logarithm of the odds (LOD) score of more than 3 were identified (Broman et al., 2003). A *Fitqtl* model calculated the percent variance explained (PVE) by the detected QTLs.

### 2.6 Identification of candidate genes and expression analysis

For the seven QTLs that mapped to a physical region smaller than 3.55 Mb, we identified the underlying candidate genes. The genes present in each of the QTL‐containing region were identified from the IWGSC high‐confidence wheat gene annotation version *2* (https://www.wheatgenome.org/). The predicted protein sequence of the identified candidate gene(s) was obtained from the IWGSC high‐confidence peptide sequence and was evaluated to identify the domains and motifs using the *Batch CD‐search* program (Lu et al., 2020) and the *InterPro Scan program* (Jones et al., 2014) (https://www.ncbi.nlm.nih.gov/Structure/bwrpsb/bwrpsb.cgi and https://www.ebi.ac.uk/interpro/search/sequence/). True orthologs (Dhaliwal et al., 2014) from the rice and Arabidopsis were identified for the candidate genes carrying domains or motifs regulating plant processes likely involved in heat stress response to validate their putative function. *Tblastn* program available at rice annotation project (http://rice.uga.edu/) and the TAIR database (https://www.arabidopsis.org/) was used for this purpose.

For the candidate genes containing domains and motifs likely to be involved in controlling heat stress tolerance, gene expression was studied using the wheat gene expression database (http://www.wheat‐expression.com/). To further shortlist the putative candidate genes, the transcript abundance of the candidate genes was studied in the target tissue. For example, to identify the candidate genes controlling TN, the transcript abundance in the shoot apical meristem was studied. Similarly, to identify the candidate gene for GY, the transcript abundance in the spike during grain filling was considered. Expression in other tissues was used as controls.

## 3 RESULTS

### 3.1 Response of the two parents to HNT stress

**FIGURE 1:**
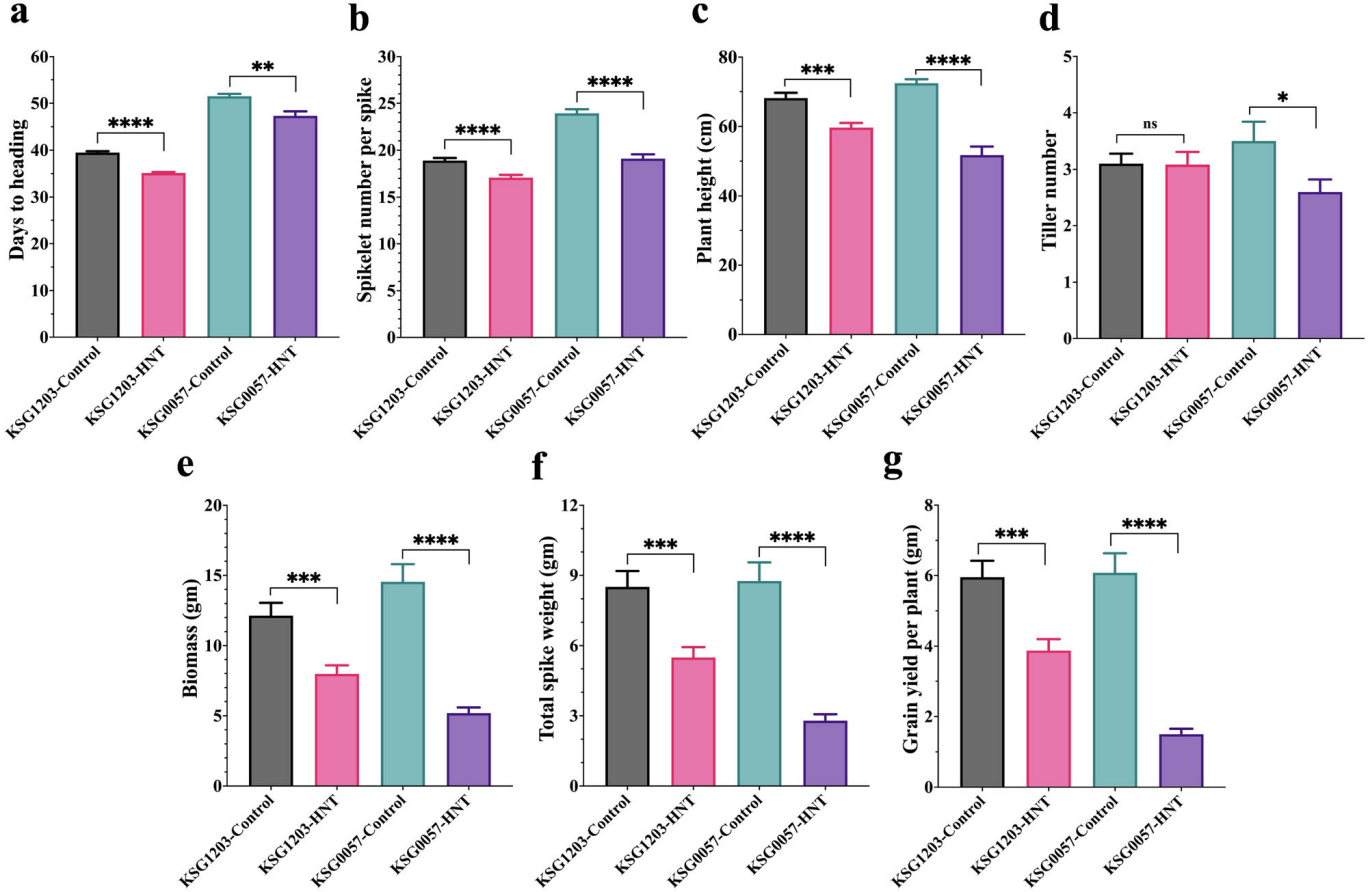
Bar plots showing phenotypic data of seven agronomic traits collected on the parents KSG1203 and KSG0057 of the doubled haploid population. The data were collected on 24 plants for each parent, evaluated under high night‐time temperature (HNT) stress in comparison to the plants grown under control conditions. The *X*‐axis represents the mean values for the genotypes under the two growth conditions and the *Y*‐axis shows values for the agronomic traits. The parent KSG1203 is shown in pink and gray colors, and KSG0057 is represented by green and purple colors. ^ns^ *p* > 0.05, ^*^ *p* < 0.05, ^**^ *p* < 0.01, ^***^ *p* < 0.001, ^****^ *p* < 0.0001. The details of statistical information are given in Table S1: (a) days to heading; (b) spikelet number per spike; (c) plant height; (d) tiller number; (e) biomass; (f) total spike weight; and (g) grain yield per plant.

**FIGURE 2:**
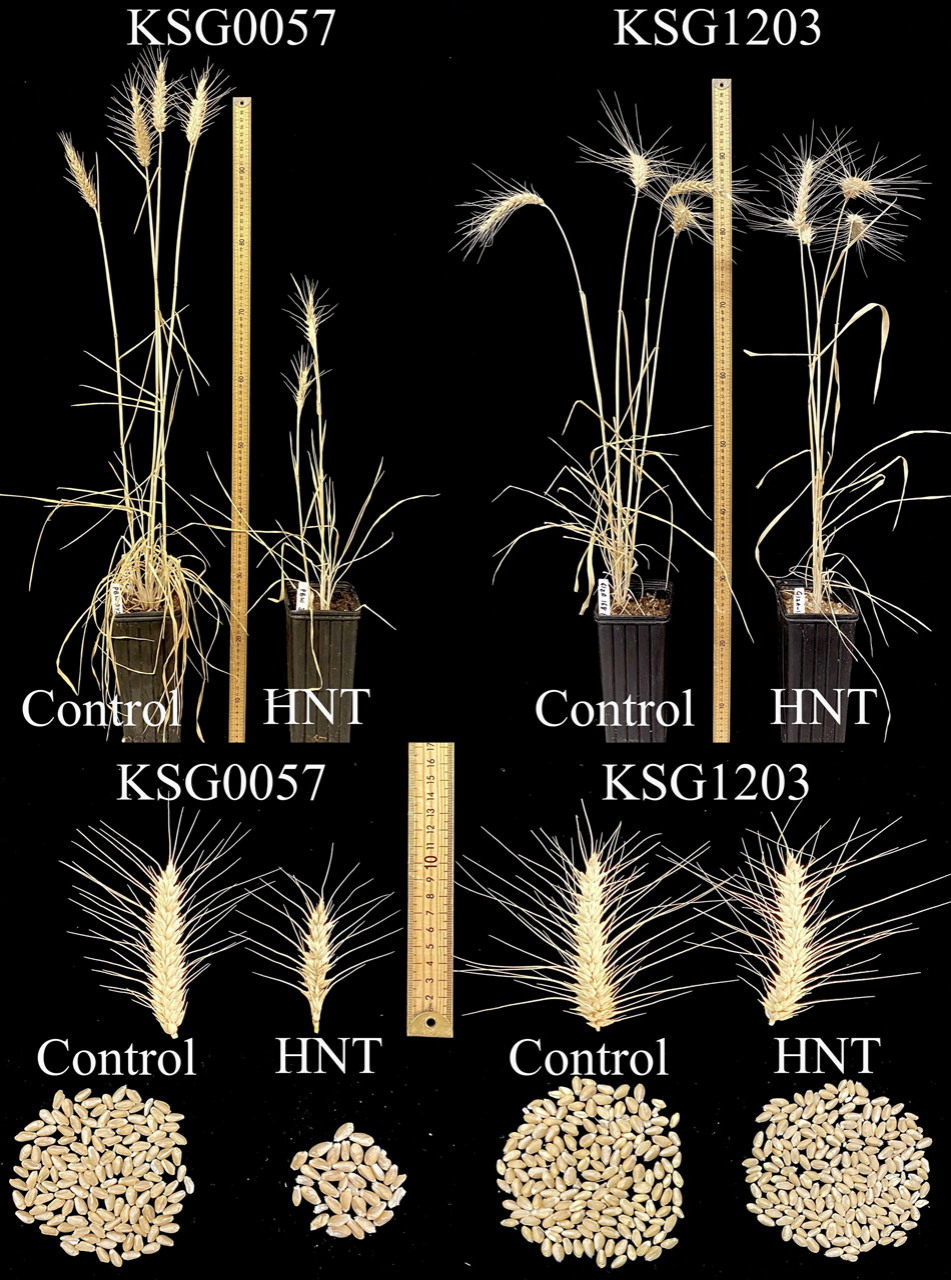
Effect of high night‐time temperature (HNT) stress on various agronomic traits (plant height, spike length and morphology, and grain number per plant) on KSG0057 and KSG1203, the two parents of the doubled haploid population.

Data of the two parents on the seven agronomic traits, that is, DTH, SN, PH, TN, total aboveground BM, TSW, and GY under HNT stress were individually compared to that under normal growth conditions to study the response of each parent to HNT stress. Relative to the control, both genotypes showed a significant reduction in DTH, with KSG1203 and KSG0057 showing 10.9% (*p*‐value < 0.0001) and 8.09% (*p*‐value = 0.0012) reduction, respectively (Figure 1 and Table S1). KSG1203 showed only 12.44% reduction in PH compared to 28.53% in KSG0057. The TN in KSG1203 showed no significant differences between HNT stress and control, whereas, in KSG0057, an ∼26% reduction was observed. The two parents also differed significantly in their response to HNT stress for SN. KSG1203 showed only ∼9% reduction compared to ∼20% for KSG0057. The trend of higher reduction in KSG0057 compared to KSG1203 was also consistent for TSW, BM, and GY (Figures 1 and 2, and Table S1).

### 3.2 Response of the DH Population to HNT stress

**FIGURE 3:**
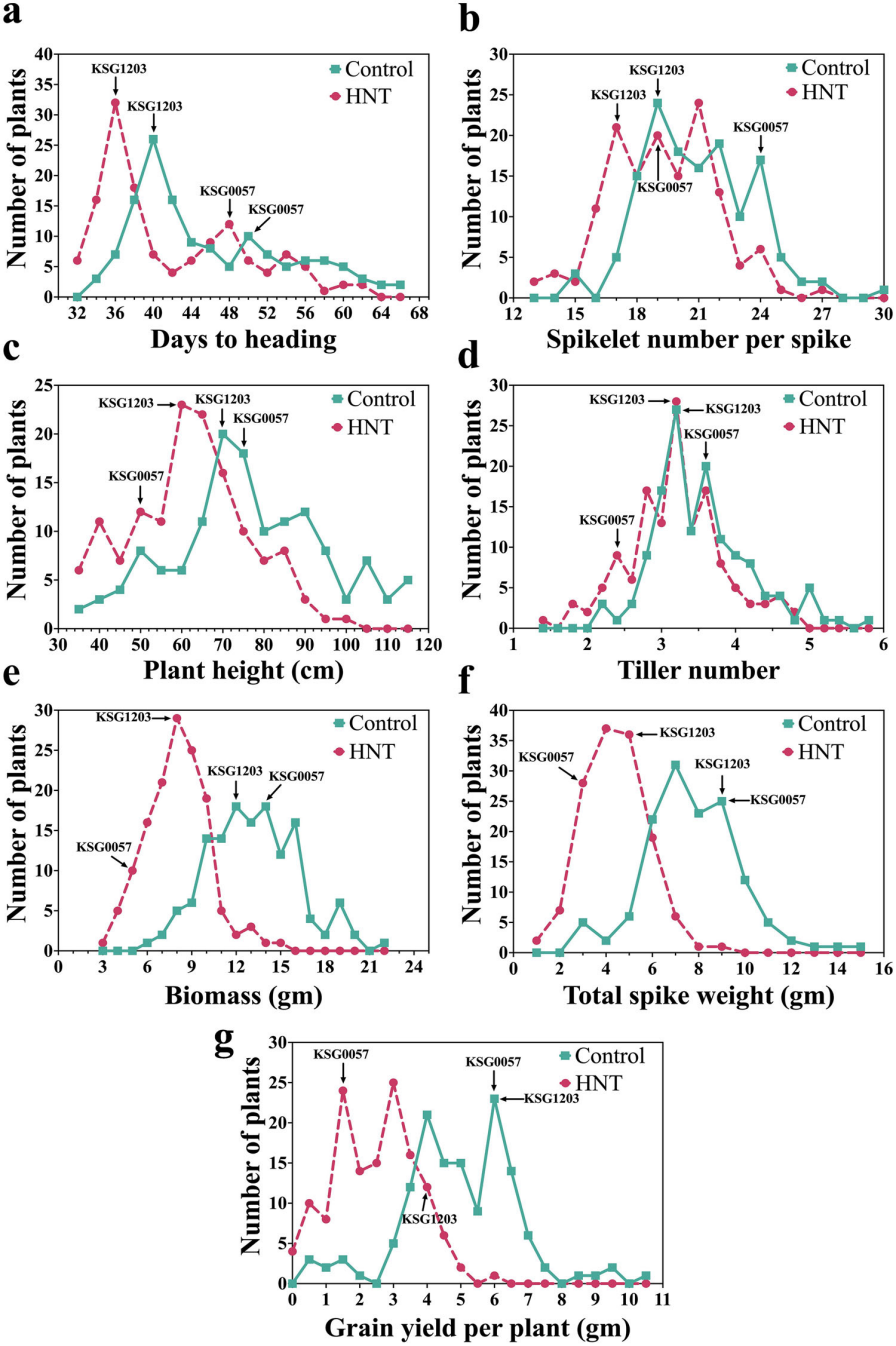
Frequency distribution of 136 doubled haploid (DH) lines under control and high night‐time temperature (HNT) stress. *Y*‐axis highlights the number of plants, whereas *X*‐axis highlights the agronomic trait. Green and Pink trend line denotes control and HNT stress, respectively. The number of plants in each category are highlighted by square for control and circle for HNT stress. Mean performance of the DH parent lines (KSG1203 and KSG0057) is also marked on the graph: (a) days to heading; (b) spikelet number per spike; (c) plant height; (d) tiller number; (e) biomass; (f) total spike weight; and (g) grain yield per plant.

**FIGURE 4:**
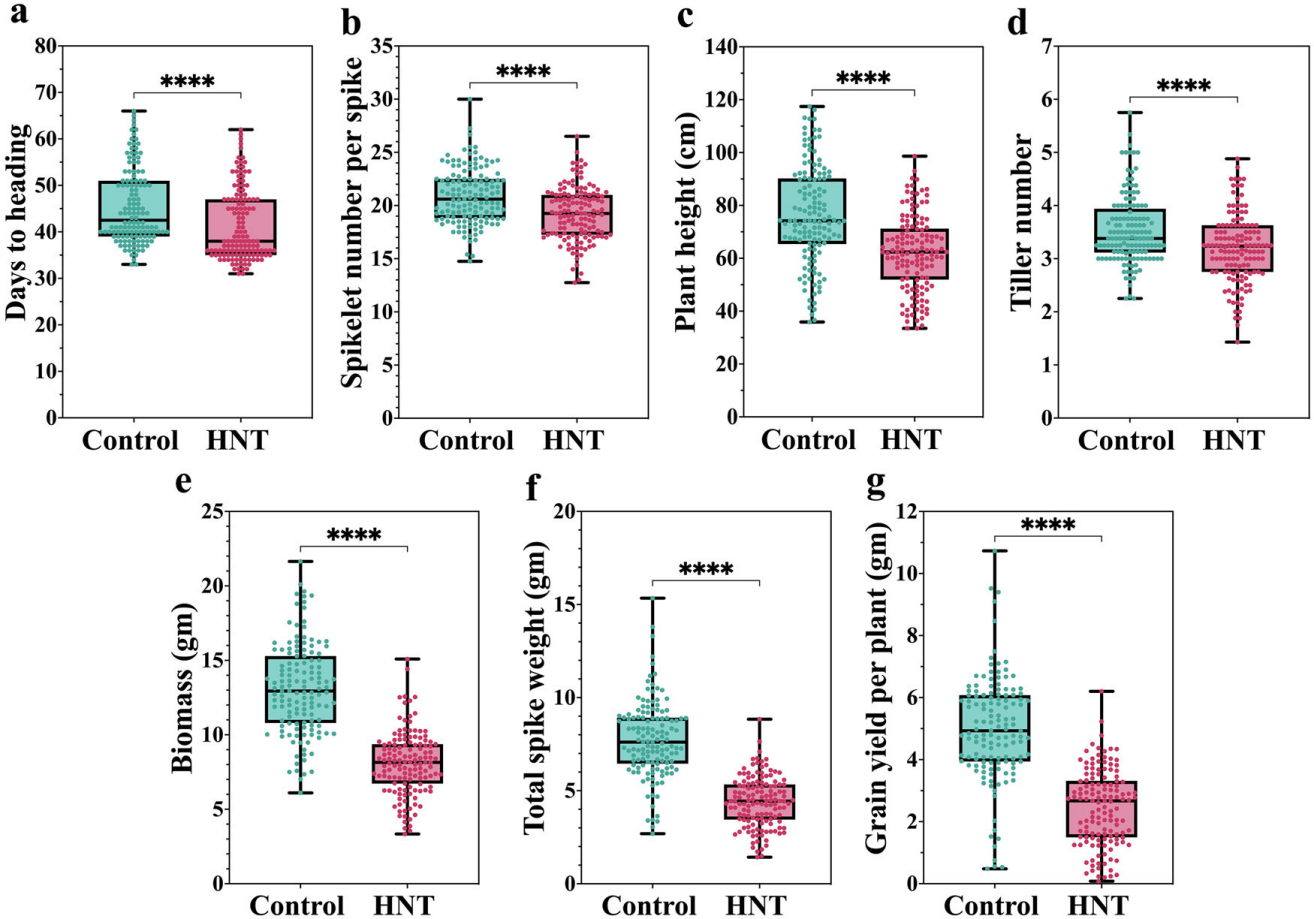
Box plots showing phenotypic data of seven agronomic traits collected on the doubled‐haploid (DH) population from a cross between KSG0057 X KSG1203. The data were collected on eight plants for each of the 136 DH plants, evaluated under high night‐time temperature (HNT) stress in comparison to the plants grown under control conditions. The *X*‐axis represents the two growth conditions and the *Y*‐axis shows mean values and range for the agronomic traits. Green‐colored boxes highlight the average performance of the DH population under control conditions, with individual dots representing individual DH lines. Similarly, pink‐colored boxes highlight the average performance of the DH population under high night‐time temperature conditions, with individual dots representing individual DH lines. Mean of each parent is given in Table S1. ^ns^ *p* > 0.05, ^*^ *p* < 0.05, ^**^ *p* < 0.01, ^***^ *p* < 0.001, ^****^ *p* < 0.0001. The details of statistical information are given in Table S1. (a) days to heading; (b) spikelet number per spike; (c) plant height; (d) tiller number; (e) biomass; (f) total spike weight; and (g) grain yield per plant.

The DH population showed normal distribution for most of the traits, although significant differences were observed between the two treatments and among different traits (Figure 3). A normal distribution was observed for SN, PH, TN, BM, TSW, and GY under control and in the HNT stress conditions, whereas a lognormal distribution was observed for DTH (Figure 3). With a range of 31–62 days, the DTH among DH lines was slightly faster under HNT stress as compared to that under control conditions, which showed a range of 33–66 days (Figure 3 and Table 1). Similarly, spikelet number per strike (SN) among DH lines was less (ranged 12.75–26.50) than that under normal conditions (a range of 14.75–30) (Figure 3 and Table 1). Similarly, the PH was also less under HNT stress conditions, with a range of 33.50–98.63 cm as compared to 35.86–117.4 cm under normal conditions (Figure 3 and Table 1). A very similar trend was observed for other traits as well. For TN, the average productive tillers ranged between 2.25 to 5.75 and 1.43 to 4.88 under normal and HNT stress conditions, respectively (Figure 3 and Table 1). For BM, the average total plant weight ranged from 6.10 to 21.64 g and 3.34 to 15.09 g under normal and HNT stress conditions, respectively. Similarly, for TSW, the average weight ranged from 1.43 to 8.84 g under HNT stress conditions compared to from 2.69 to 15.34 g under control conditions. For GY, the effect of HNT stress was dramatic. The average GY ranged from 0.08 to 6.20 g under HNT stress conditions compared to from 0.48 to 10.73 g under control conditions (Figure 4 and Table 1).

**TABLE 1:** Average performance of seven agronomic traits under control and high night‐time temperature (HNT) stress conditions in 136 doubled haploid (DH) lines.

Trait	Control		HNT		
	Average	Range	Average	Range	RP%^a^
Days to heading	45.4	33–66	41.41	31–62	8.79
Spikelet number	20.87	14.75–30.0	19.22	12.75–26.50	7.91
Plant height (cm)	76.14	35.86–117.4	62.04	33.50–98.63	18.52
Tiller number	3.538	2.25–5.75	3.194	1.43–4.88	9.72
Biomass (g)	13.11	6.10–21.64	8.083	3.34–15.09	38.34
Total spike weight (g)	7.746	2.69–15.34	4.405	1.43–8.84	43.13
Grain yield per plant (g)	4.975	0.48–10.73	2.458	0.08–6.20	50.59

^a^ RP%: Relative performance percentage or percent reduction.

Pearson correlation coefficient was calculated to study the correlation among different agronomic traits, and the data are given in Figure S1. Under the control conditions, GY showed a very high positive correlation with TSW (*r* = 0.95), moderate positive correlation with BM (*r* = 0.61) and TN (*r* = 0.42), and low correlation with SN (*r* = 0.19). However, GY showed low negative or no correlation with PH (*r* = −0.13) and DTH (*r* = −0.02). Interestingly, SN showed a strong positive correlation with DTH (*r* = 0.78). The correlation values for various agronomic traits were similar under control and HNT stress conditions. GY showed a high positive correlation with TSW (0.92) and a moderate correlation with BM (0.45) and TN (0.33). The key difference between control and HNT stress was for the correlation between GY and DTH, which was negative (−0.38) under stress conditions but essentially nonexistent (r = −0.02) under normal conditions.

Linear regression analysis was performed by considering GY as a dependent variable and PH, DTH, SN, BM, TSW, and TN as independent variables (Figure S2). BM explained ∼37% variation in GY under control conditions (*R* ^2^ = 0.3692, *p* < 0.0001), which dropped to 20% under HNT stress (*R* ^2^ = 0.2036, *p* < 0.0001). TSW explained ∼91% of the variation in GY under control conditions (*R* ^2 ^= 0.9077, *p* < 0.0001) and ∼85% under HNT stress (*R* ^2^ = 0.8490, *p* < 0.0001). TN explained only ∼18% of the variation in GY under control conditions (*R* ^2 ^= 0.1763, *p* < 0.0001), which was even less under HNT stress (*R* ^2^ = 0.1082, *p* < 0.0001). PH did not explain any variation in GY under control or HNT conditions. As seen for correlations, DTH was relevant for GY only under HNT stress (*R* ^2^ = 0.1477, *p* < 0.0001) and not under control conditions (*R* ^2^ = 0.0004238, *p* = 0.8141). Similarly, SN did not have much effect on GY as it explained only ∼3% of the variation under control conditions (*R* ^2 ^= 0.03499, *p* = 0.0305) and 0.6% under HNT stress conditions (*R* ^2^ = 0.006181, *p* = 0.3629).

### 3.3 QTL analysis under normal and HNT stress conditions

**FIGURE 5:**
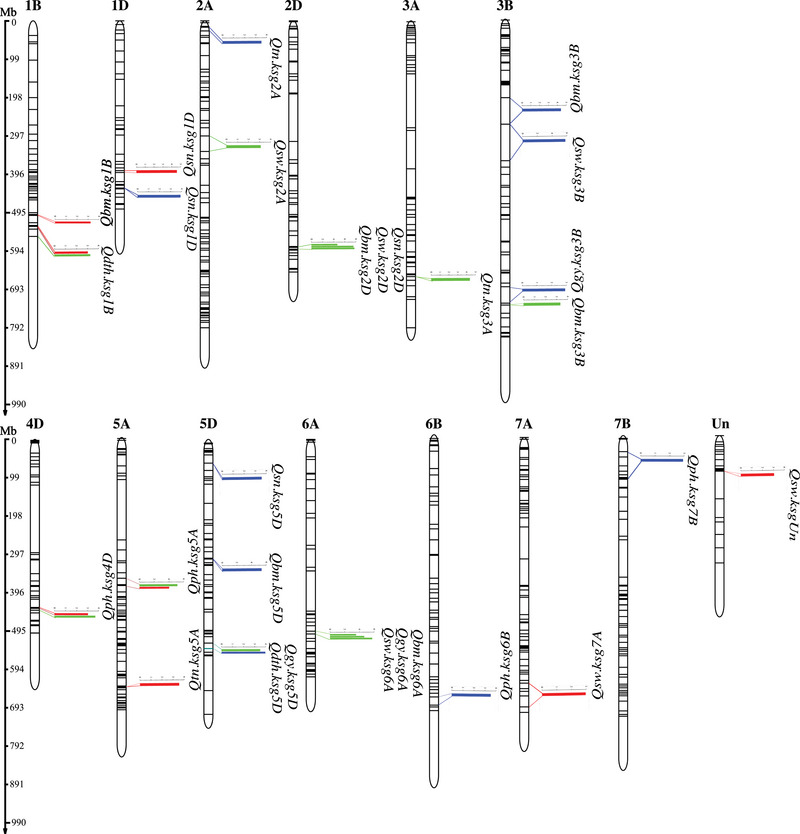
Physical map of quantitative trait loci (QTLs) associated with seven agronomic traits in a doubled haploid (DH) population evaluated under control and high night‐time temperature (HNT) stress conditions. The IWGSC RefSeq v1.0 physical map position (Mb) is shown on the left side of the chromosome, with each horizontal line representing a single nucleotide polymorphism (SNP) marker. QTL intervals are shown on the right side of each chromosome, with bars indicating their individual LOD value and color‐coded flanking markers highlighting the QTL intervals. QTLs associated with control, HNT, and heat susceptibility index (HSI) treatments are marked in green, red, and blue colors, respectively.

Composite interval mapping was performed using the mean phenotypic data for the seven agronomic traits. Three types of phenotypic data were used, that is, mean trait values under normal and HNT stress conditions and percentage reduction between control and HNT stress conditions. SBG of the DH population was performed to generate 5687 physically anchored SNPs representing 1154 genes. A total of 32 QTLs mapping to 25 unique intervals on 13 of the 21 wheat chromosomes were identified for the seven phenotypic traits (Figure 5 and Table 2). The maximum number of QTLs per chromosome was four, each on chromosomes 1B, 3B, and 5D. Chromosomes 3A, 6B, 7A, and 7B showed only one QTL each (Figure 5). One of the 32 QTLs mapped on the unanchored (Un) part of the wheat genome. At an LOD score of 3 or above, 13 of the 32 QTLs were for the traits measured under control conditions, eight for HNT stress, and 11 QTLs were identified for the percentage reduction under HNT stress (heat susceptibility index [HSI]) (Table 2). The QTL for PH on chromosomes 4D and 5A was detected both under control and HNT stress conditions. Similarly, the QTLs for DTH and GY on chromosome 5D were detected both under control and HSI.

**TABLE 2:** Summary of the 25 unique intervals containing 32 quantitative trait loci (QTLs) associated with seven traits evaluated using composite interval mapping (CIM) under control, high‐night temperature (HNT) stress, and heat susceptibility index (HSI).

QTL name	Treatment	Trait	Chr	Position (Mb)	LOD score	Left flanking marker	Right flanking marker	PVE (%)	favorable Allele
Qdth.ksg1B	Control	DTH	1B	539.7	3.252	TraesCS1B02G315300.2_650	TraesCS1B02G332500.1_726	8.145788	KSG0057
Qsw.ksg2A	Control	TSW	2A	338.5	3.101	TraesCS2A02G243000.1_989	TraesCS2A02G248500.1_125	7.860168	KSG1203
Qbm.ksg2D	Control	BM	2D	586.8	7.54	TraesCS2D02G486100.1_440	TraesCS2D02G495300.1_376	9.121407	KSG1203
Qsw.ksg2D	Control	TSW	2D	586.8	7.734	TraesCS2D02G482300.1_1993	TraesCS2D02G486100.1_440	9.399943	KSG1203
Qsn.ksg2D	Control	SN	2D	586.8	4.649	TraesCS2D02G486100.1_440	TraesCS2D02G495300.1_376	8.078064	KSG1203
Qtn.ksg3A	Control	TN	3A	661.8	4.363	TraesCS3A02G420000.1_99	TraesCS3A02G421400.2_267	4.182008	KSG1203
Qbm.ksg3B	Control	BM	3B	740.5	3.33	TraesCS3B02G495300.1_1679	TraesCS3B02G497200.1_570	3.057264	KSG0057
Qph.ksg4D	Control	PH	4D	437.52	3.687	TraesCS4D02G266700.2_343	TraesCS4D02G270500.1_108	7.069815	KSG1203
Qph.ksg5A	Control	PH	5A	481.66	5.132	TraesCS5A02G270900.1_1055	TraesCS5A02G184400.1_181	1.334012	KSG0057
Qdth.ksg5D	Control	DTH	5D	526.9	3.508	TraesCS5D02G495600.1_132	TraesCS5D02G521900.1_477	4.864513	KSG1203
Qgy.ksg6A	Control	GY	6A	503.5	5.727	TraesCS6A02G270300.1_130	TraesCS6A02G276400.1_941	6.116546	KSG0057
Qsw.ksg6A	Control	TSW	6A	503.5	4.641	TraesCS6A02G270300.1_130	TraesCS6A02G276400.1_941	6.031989	KSG0057
Qbm.ksg6A	Control	BM	6A	503.5	3.538	TraesCS6A02G270300.1_130	TraesCS6A02G276400.1_946	3.337388	KSG0057
Qdth.ksg1B	HSI	DTH	1B	38.1	2.976	TraesCS1B02G376500.1_481	TraesCS1B02G055300.2_2882	4.398291	KSG1203
Qsn.ksg1D	HSI	SN	1D	431.7	3.89	TraesCS1D02G341600.1_466	TraesCS1D02G342100.1_1761	4.145012	KSG0057
Qtn.ksg2A	HSI	TN	2A	15.6	3.525	TraesCS2A02G036200.1_919	TraesCS2A02G041800.1_1278	7.895703	KSG1203
Qbm.ksg3B	HSI	BM	3B	270.9	4.392	TraesCS3B02G191100.2_262	TraesCS3B02G220700.1_631	9.059314	KSG1203
Qsw.ksg3B	HSI	TSW	3B	270.89	5.785	TraesCS3B02G220700.1_632	TraesCS3B02G234100.1_237	10.48549	KSG1203
Qgy.ksg3B	HSI	GY	3B	693.3	2.91	TraesCS3B02G452200.1_1678	TraesCS3B02G489900.1_343	8.12601	KSG1203
Qsn.ksg5D	HSI	SN	5D	60.5	3.582	TraesCS5D02G040200.1_2805	TraesCS5D02G065100.3_208	8.348787	KSG1203
Qbm.ksg5D	HSI	BM	5D	309	5.356	TraesCS5D02G203800.1_749	TraesCS5D02G205100.1_2290	4.450182	KSG0057
Qgy.ksg5D	HSI	GY	5D	526.9	3.968	TraesCS5D02G495600.1_132	TraesCS5D02G521900.1_477	4.221128	KSG1203
Qph.ksg6B	HSI	PH	6B	699	3.526	TraesCS6B02G403700.1_884	TraesCS6B02G431200.1_531	4.630929	KSG0057
Qph.ksg7B	HSI	PH	7B	43.2	2.922	TraesCS7B02G043400.1_486	TraesCS7B02G096600.1_358	9.242318	KSG1203
Qbm.ksg1B	HNT	BM	1B	500.54	3.223	TraesCS1B02G287800.1_1539	TraesCS1B02G289200.1_1058	4.083547	KSG0057
Qdth.ksg1B	HNT	DTH	1B	539.66	3.036	TraesCS1B02G309600.1_474	TraesCS1B02G315300.2_650	7.798185	KSG1203
Qsn.ksg1D	HNT	SN	1D	388.6	4.575	TraesCS1D02G290400.1_236	TraesCS1D02G292700.1_1005	5.181862	KSG0057
Qph.ksg4D	HNT	PH	4D	437.5171	3.03	TraesCS4D02G264200.1_583	TraesCS4D02G266700.2_343	6.677678	KSG1203
Qph.ksg5A	HNT	PH	5A	481.66	4.016	TraesCS5A02G270900.1_1055	TraesCS5A02G184400.1_181	4.734565	KSG0057
Qtn.ksg5A	HNT	TN	5A	645.9	3.55	TraesCS5A02G468600.1_454	TraesCS5A02G468700.1_717	1.817731	KSG0057
Qsw.ksg7A	HNT	TSW	7A	636.2	2.964	TraesCS7A02G442200.1_508	TraesCS7A02G510800.1_1747	8.987034	KSG1203
Qsw.ksgUn	HNT	TSW	Un	93.4	3.052	TraesCSU02G269500.1_383	TraesCSU02G110400.1_810	9.331334	KSG1203

*Note*: The wheat gene nomenclature requires using italics for genes and QTL names. The chromosome and physical map are based on the International Wheat Genome Sequencing Consortium (IWGSC) Ref Seq 1.0.

Abbreviations: LOD, logarithm of the odds; PVE (%), percent variance explained.

Among traits, the maximum number of QTLs was six, which were detected for PH, TSW, and BM. DTH and SN showed four QTLs each, whereas the number of QTLs for TN and GY was three. Two QTLs were detected for PH each under control, HNT stress, and for HSI (Table 2 and Figure 5). Similarly, the three QTLs for TN were detected under all three conditions. Out of the four QTLs for DTH, two were identified under control conditions, and one each for HNT and HSI. Similarly, for four SN QTLs, two were identified for HSI, one under control, and one under HNT. Out of the six QTLs for TSW, three were detected under control conditions, two under HNT, and one under HSI. More QTLs for BM (three) were identified under control conditions in comparison to that for HNT and HSI (Table 2). No QTL for GY was detected under HNT, whereas two QTLs were detected under HSI and one under control (Table 2).

Distribution of QTLs on chromosomes was variable among traits. Three of the four QTLs for DTH were present on chromosome 1B, and the fourth was present on 5D (Table 2 and Figure 5). The six QTLs for PH were distributed on four different homoeologous groups (4D (2), 5A (2), 6B, and 7B). All three QTLs for TN were present on the A genome chromosomes (2A, 3A, and 5A), whereas the QTLs for SN were all present on the D genome chromosomes [1D (2), 2D (1), and 5D (1)]. The six QTLs for the TSW and BM traits were present on different chromosomes of the three genomes except for BM showing two QTLs on chromosome 3B. Similarly, the three QTLs for GY mapped on three different chromosomes of the three genomes (Table 2 and Figure 5).

Size of the QTL containing intervals ranged from 21.3 kb to 97.48 Mb (Table S2). The QTLs for TN mapped to the smallest segments. QTL (*Qtn.ksg5A*) for TN on chromosome 5A mapped to a 21.3 kb region and that on 3A (*Qtn.ksg3A*) to a 1.134 Mb region. Similarly, the interval size for two of the four QTLs on chromosome 1D for SN was also small with 0.124 Mb for *Qsn.ksg1D* and 3.23 Mb for *Qsn.ksg1D*. Three of the QTLs for BM also mapped to small chromosomal regions with 3.55 Mb for *Qbm.ksg1B*, 1.68 Mb for *Qbm.ksg5D*, and 1.2 Mb for *Qbm.ksg3B*. Overall, size of nine QTL containing regions was smaller than 5 Mb and 17 were smaller than 20 Mb (Table S2).

The percent phenotypic varianceexplained by the individual QTLs ranged from 2% to 10%, whereas combined phenotypic variance explained for each trait ranged from 4.08% for BM under HNT stress to 23.29% for TSW under control conditions. The highest level of PVE explained by QTLs was for TSW where individually it ranged from 6% to 10% and combined from 10.49% to 23.29%. The lowest level of PVE explained was for TN where the range for individual effect was from 1.8% to 7.9%. Of the 19 QTLs mapped for night‐heat stress tolerance, the QTLs with the highest phenotypic variance were for DTH located on chromosome 1B, TN on 2A, and for PH on 7B explaining 7.8%, 7.9%, and 9.24% of the variation, respectively (Table 2). Similarly, for SN and TSW, the highest phenotypic variance of 8.3% and 10.5% was explained by the QTLs mapped on chromosomes 5D and 3B, respectively (Table 2). Furthermore, for BM and GY, the highest phenotypic variance of 9.1% and 8.1% was explained by the QTLs mapped on chromosome 3B (Table 2).

### 3.4 Candidate genes underlying the QTLs

Physical size of each of the intervals used for the QTL mapping was determined using the IWGSC reference genome version 2 (see Section 2). With 5687 markers, the wheat genome was divided into 5708 intervals. The size range of the 25 QTL‐containing intervals ranged from 21.3 kb to 97.48 Mb (Table S2). The number of candidate genes in each of the QTL‐containing interval was counted using the IWGSC high‐confidence gene annotation version 1.1 (The International Wheat Genome Sequencing Consortium [IWGSC] et al., 2018). A total of 4338 high‐confidence genes were identified in the 25 intervals containing 32 QTLs. The number of candidate genes in each of the 25 intervals ranged from 2 to 867. The smallest interval containing a QTL for TN under HNT stress (*Qtn.ksg5A*) had only two genes. Similarly, the interval containing QTL for SN under HSI (*Qsn.ksg1D*) had only seven genes (Table S2).

Genes from the regions containing *Qbm.ksg1B*, *Qbm.ksg5D*, *Qsn.ksg1D*, *Qsn.ksg1D*, *Qtn.ksg5A*, and *Qph.ksg4D*, each of which had less than 30 genes, were analyzed for specific domain and motif that are expected to be present in the corresponding gene controlling the trait. This analysis was done using the NCBI Batch CD‐search program (https://www.ncbi.nlm.nih.gov/Structure/bwrpsb/bwrpsb.cgi) and the results are given in Table 3 and Table S3. The candidate genes were selected based on the presence of functional domain/motif, previously characterized for role in heat stress tolerance, previously characterized for role in HNT stress tolerance, target tissue‐specific gene expression, or positive gene expression in response to heat stress. For the smallest QTL interval controlling TN under HNT stress (*Qtn.ksg5A*), two genes were identified. Domain‐motif analysis revealed that one of these genes contained KH‐I and the other contained RplQ domain (Table S3). Additional characterization of the genes using literature, gene expression, and its orthologs in rice and Arabidopsis revealed the KH‐I domain‐containing gene (TraesCS5A02G468700.1) is a likely candidate for the trait due to its putative role in transcription regulation and higher transcript abundance in the target tissue (Table 3).

**TABLE 3:** Putative candidate genes from the smallest quantitative trait locus (QTL) intervals controlling night‐heat specific response for four agronomic traits (biomass, spikelet number [SN], tiller number [TN], and plant height [PH]).

Putative candidate gene	Trait	Domain/motif	Expression	HS	NH	Putative function
TraesCS1B02G288000.1	Biomass	β‐Glucosidase	+	+	+	Cellulose metabolism
TraesCS1B02G289100.1	Biomass	Ankyrin repeat	+	+	NA	Interact with Hsp90/Hsp70 as co‐chaperones
TraesCS1D02G342000.1	SN	WD40 repeat	+	+	NA	Transcription regulation
TraesCS1D02G342100.1	SN	ClpA	+	+	NA	Heat‐shock protein
TraesCS5A02G468700.1	TN	KH‐I domain	+	+	NA	Transcription regulation
TraesCS1D02G292200.1	SN	B3_domain	+	+	NA	Vegetative to flowering transition
TraesCS1D02G292700.1	SN	WRKY	+	+	NA	DNA‐binding
TraesCS4D02G266400.1	PH	GRAS	+	+	+	GA signaling/stem elongation

*Note*: Putative candidate genes were identified using domain/motif analysis, putative function of orthologs in Arabidopsis and rice, gene expression, and evidence in night heat‐specific physiological processes.

Abbreviations: Expression, target tissue‐specific gene expression; GA, gibberllic acid; HS, previously characterized for role in heat stress tolerance; NA, not available; NH, previously characterized for role in night‐heat stress tolerance; +: positive gene expression or role in heat stress response.

For the seven genes present in the QTL interval (*Qsn.ksg1D*) controlling spikelet number under night heat, the domain‐motif analysis revealed diverse functional domains ranging from mitogen‐activated protein kinase to heat shock domain‐containing genes (Table S3). Further characterization of the genes using literature, gene expression, and orthologs in rice and Arabidopsis revealed two putative candidate genes, that is, WD40 domain‐containing gene (TraesCS1D02G342000.1) and heat shock domain‐containing gene (TraesCS1D02G342100.1), both with a known function in abiotic stress tolerance (Table 3). Additional functional annotation of the WD40 domain‐containing genes using its functional ortholog in Arabidopsis revealed a known function in RNA processing and female gametophyte development (Li et al., 2010).

A similar analysis of the 25 genes present in the QTL interval (*Qsn.ksg1D*) controlling SN under HNT stress revealed a diverse family of functional domains including a major superfamily facilitator and plant‐specific B3‐DNA binding domains (Table S3). One of these genes (TraesCS1D02G292700.1) has a DNA‐binding domain with a known function in heat‐stress tolerance and gene expression in the target tissue. Similarly, another candidate gene (TraesCS1D02G292200.1) carrying plant‐specific B3‐DNA binding domain controlling vegetative to flowering transition was identified (Table 3).

For the QTL interval (*Qph.ksg4D*) controlling PH under HNT stress that has 27 genes, domain‐motif analysis revealed genes carrying functional domains and motifs including the GRAS domain family and amino acid transporter (Table S3). Further characterization of the genes as mentioned above identified a candidate gene (TraesCS4D02G266400.1) with a known function in regulating stem elongation during night‐heat stress (Table 3). Similarly, QTL interval (*Qbm.ksg1B*) controlling BM had 16 genes (Table S3). Domain and motif analysis revealed candidate genes with putative functions including signal transduction and carbohydrate transport. One of these candidate genes (TraesCS1B02G288000.1) has a role in cellulose metabolism and night‐heat stress response (Table 3). Similarly, another candidate gene (TraesCS1B02G289100.1) was identified that contained *Ankyrin* repeat with a known function in heat‐stress tolerance through interaction with Hsp90/Hsp70 co‐chaperons (Table 3).

### 3.5 Selection of high‐yielding DH lines for HNT stress tolerance

With the objective to identify DH lines showing agronomic potential better than the parents, the DH lines were evaluated for GY both under normal as well as under HNT stress (Tables S4 and S5). The two parents did not show much difference in yield under normal conditions. The 136 DH lines showed significant variation for single plant yield with 32 lines showing GY higher than that of KSG0057 and 37 showed higher than KSG1203. Correspondingly, 102 lines showed yield less than KSG0057 and 98 less than KSG1203. Under HNT stress, 101 DH lines showed yield higher than KSG0057 and 18 higher than KSG1203.

Under normal conditions, the DH151 showed the highest GY that was 43.3%–44.5% higher than the two parents. Under HNT stress, the heat stress tolerant parent KSG1203 yielded 61.2% higher than the mega variety KSG0057. The DH52 was the highest yielding under HNT stress with ∼75.8% higher yield than KSG0057. Its yield was ∼37.6% higher than that of KSG1203. Additionally, the yield numbers for the second ranked line DH63 were similar to that of DH52. Yield of these two lines under normal conditions was 4.5%–6.6% higher than the two parents.

## 4 DISCUSSION

### 4.1 Drastic effect of HNT stress on agronomic performance of mega variety PBW343

The semidwarf female parent cultivar PBW343 (KSG0057 is a selection out of PBW343) with pedigree: Nord Desprez/VG9144//Kalyan Sona/Bluebird 3/Yaco/4/Veery#5, was developed by CIMMYT, Mexico. It was released in 1995 under different names in various South East Asian countries. In India, it was released under the name of PBW343. By 2002–2003, just in India, it was cultivated on 7.28 million ha. Its popularity in other countries in the region was similar. In the current study, we have shown it to be severely sensitive to HNT stress as its yield was ∼75% lower as compared to that under the control conditions. The other agronomic traits including SN, TN, PH, BM, and TSW were also severely impacted by HNT stress in this cultivar (Figures 1 and 2 and Table S1). These findings show vulnerability of currently popular varieties to HNT stress, which is expected to become worse in the near future due to climate change. This is possibly because no selection for HNT stress tolerance is made in any of the wheat breeding programs. The results and markers developed during the study will make it possible to breed for wheat varieties tolerant to HNT stress.

On the other hand, the semidwarf male parent cultivar KSG1203 with the pedigree: MRL/BUC/Seri.CM93046‐8 M‐0Y‐0 M‐2Y‐0B‐0GZ, was released in Egypt in 1999. It was selected out of ∼2000 carefully selected wheat lines for different types of heat stress tolerance including that for HNT stress (see Section 2). As a result, it turned out to be considerably tolerant to HNT stress as it showed only ∼35% reduction in yield as compared to ∼75% in KSG0057 (Figures 1 and 2 and Table S1). These results demonstrate the success of our selection for heat stress tolerance in wheat.

### 4.2 Effect of HNT stress on various agronomic traits

Comparison with the controlled condition data, the night heat stress negatively impacted all seven agronomic traits although there was dramatic difference between the tolerant and the susceptible line for the extent of impact. The percentage reduction in the trait value ranged from ∼0.5% to 35% in the tolerant parent (KSG1203) as compared to from 8.1% to 75.3% in the susceptible parent (KSG0057). Additionally, a comparison of the parental genotypes with the day‐time heat stress screening data (S. Kumar et al., 2024) suggested that the TN trait in KSG1203 is differentially regulated by day‐time and night‐time heat stress. In rice, HNT stress at 28°C resulted in ∼30% reduction in tiller/panicle development of a susceptible parent (A. Kumar, Gupta, et al., 2021). However, the response was different in the tolerant parent, that is, 23% reduction in TN during daytime heat stress and no reduction during night‐time heat stress, suggesting that the underlying physiological pathways impacted by day‐ and night‐time heat stress to regulate tillering might be unique. Similarly, night heat stress significantly reduced PH and the extent of reduction was significantly different between the tolerant and the susceptible lines (Figure 3). Similar reduction in stem elongation in response to night‐time temperature stress was also observed in tomato, thus it might be a common effect among plants (Ohtaka et al., 2020). The most drastic effect of HNT stress was on BM, TSW, and GY (Figures 3 and 4). Since these three are interlinked traits, the genetic determinants for this effect of night heat stress might be common among these traits.

### 4.3 HNT stress causes early flowering in wheat

Under control conditions, KSG1203 flowered 13 days earlier than KSG0057 (Figure 3a). HNT stress caused early flowering in both parents, by 5 days in KSG0057 and 4 days in KSG1203 (Figure 3a). The difference for flowering time between the two parents under HNT was similar to that under control conditions (12 days). The Pearson's correlation coefficient analysis revealed that flowering time showed no correlation with GY under control conditions (Figure S1a). However, under HNT stress, flowering time showed moderately negative correlation with GY suggesting that a longer vegetative stage under HNT stress has a negative effect on GY (Figure S1b). Not observed earlier, flowering time interestingly showed strong positive correlation with spikelet number both under control and HNT, thus suggesting that genes controlling these traits are either similar or are tightly linked. If true in other backgrounds also, it might be possible to use flowering time as a morphological marker for breeding for night heat stress tolerance.

### 4.4 Transgressive segregation for traits in the DH population

The DH population showed dramatic transgressive segregation in both positive and negative direction for all seven traits although the extent was different among traits. GY and TSW under both control and HNT had more negative transgressive segregants than positive, suggesting that DH parents had fewer (<50%) favorable alleles for these traits. Thus, the probability of having more favorable allele combinations in the population was less. Additionally, more transgressive segregants means that GY and TSW is likely to be controlled by multiple genes. On the contrary, DTH, PH and TN had more positive transgressive segregants, suggesting that DH parents had more favorable alleles for these two traits. BM and DTH had equal numbers of transgressive segregants in the two directions, suggesting DH parents had about equal numbers of favorable and unfavorable alleles. SN and TN had fewer transgressive segregants than GY, PH, BM, and TSW, suggesting that these traits are likely to be controlled by fewer genes. More number of QTLs were identified for traits (PH, BM, and TSW) that showed more transgressive segregants and fewer number of QTLs for traits (SN and TN) showing fewer transgressive segregants.

### 4.5 QTL mapping for HNT stress in wheat

In our effort to map the genetic regions that regulate HNT stress tolerance, we identified 25 chromosomal intervals to be carrying 32 QTLs for the seven traits and 19 QTLs for important agronomic traits in wheat under HNT stress. QTL mapping through linkage and genome‐wide association mapping has identified genetic regions controlling heat‐stress tolerance. A meta‐analysis in wheat identified 441 QTLs (for daytime heat stress) related to 31 heat‐responsive traits (S. Kumar, Singh, et al., 2021), and another meta‐QTL analysis identified that eight major clusters on chromosomes 1B, 2B, 2D, 4A, 4B, 4D, 5A, and 7A carried eight major QTL clusters controlling drought and heat‐stress tolerance (Acuña‐Galindo et al., 2015). The availability of the wheat reference genome provides a great opportunity to advance wheat gene discovery and mapping (The International Wheat Genome Sequencing Consortium [IWGSC] et al., 2018). Physical mapping of QTLs using wheat reference genome has identified 44 QTLs in different production systems in four spring wheat populations (Semagn et al., 2021). However, there was no study focused on mapping the QTLs in wheat under high night‐time stress. Thus, this is the first study in wheat where we have established the impact of HNT stress on various agronomic traits and identified QTLs and corresponding candidate genes. We have identified 19 QTLs for HNT stress tolerance on 13 wheat chromosomes, explaining a cumulative phenotypic variance ranging from 9.72% to 28.81%. Recent genome‐wide association analysis in rice identified genetic regions explaining significant phenotypic variance for panicle length and spikelet per panicle under night‐time heat stress (A. Kumar, Gupta, et al., 2021). Additional work in Arabidopsis has revealed that the evening‐expressed clock component TOC1 regulates evening‐specific temperature stress response by regulating the temperature‐responsive transcription factors (Zhu et al., 2016). Furthermore, compared to the day‐time heat stress QTL mapping in the same population (S. Kumar et al., 2024), we are reporting that the QTLs identified in our study are unique, validating that night‐time heat stress targets unique physiological and developmental processes compared to that for daytime heat stress.

### 4.6 Candidate gene analysis for HNT stress tolerance

We further identified the corresponding candidate genes for the smallest QTLs intervals. Only a handful of candidate genes have been characterized in wheat for their role in heat‐stress tolerance in wheat (Hu et al., 2018; Xue et al., 2015; Zang et al., 2017; Zhang et al., 2017). Identification of candidate genes using a bioinformatics and comparative genomics‐based approach provides a great tool for shortlisting putative candidate genes from physically anchored QTL intervals. Our candidate gene analysis identified eight putative candidate genes for four agronomic traits under HNT stress (Table 3). Two of the candidate genes, that is, TraesCS1B02G288000.1 and TraesCS1B02G289100.1, carried β‐glucosidase and ankyrin repeats, respectively (Table 3). Cellulose synthesis and metabolism are differentially regulated by multiple abiotic stresses, including night‐time heat stress (Tian et al., 2013), and β‐glucosidase is a major player in cellulose metabolism (Singhania et al., 2013). Genome‐wide association analysis for HNT stress in rice and candidate gene analysis revealed an over‐enrichment of candidate genes from cellulose and xylene biosynthesis pathways (A. Kumar, Gupta, et al., 2021). Similarly, ankyrin repeat proteins play a critical role in plant development and their differential response to abiotic stress (Zhao et al., 2020). Similarly, candidate gene analysis for the TN QTL revealed two candidate genes, out of which one was a KH‐I domain‐containing protein (Table 3). KH‐I domain‐containing proteins are known to regulate multiple plant development processes, including branching, and have been revealed to play a crucial role in abiotic stress response (Guan et al., 2013).

Stem elongation in plants is negatively regulated by HNT stress, where gibberellic acid hormones have been identified to play a key role (Ohtaka et al., 2020). Our candidate gene analysis for the QTL controlling PH at HNT stress revealed the presence of a GRAS family domain protein (TraesCS4D02G266400.1) that is known to play a key role in GA (gibberellic acid) signaling (Yoshida et al., 2014). Additionally, four genes were identified from two QTL intervals (*Qsn.ksg1D.1* and *Qsn.ksg1D.2*) as candidates for controlling spikelet number under HNT stress (Table 3). In QTL (*Qsn.ksg1D.1*), one of the two putative candidate genes was heat‐shock protein (TraesCS1D02G342100.1), which has been functionally characterized in maize, Arabidopsis, and rice for its role in controlling heat‐stress tolerance (Frova & Gorla, 1993; Swindell et al., 2007; Wang et al., 2014). Similarly, the second candidate gene (TraesCS1D02G342000.1) carry a WD40 domain, which has been functionally characterized to regulate global transcription and play a key role in female gametophyte development in Arabidopsis (Li et al., 2010). Similarly, candidate gene analysis for QTL interval *Qsn.ksg1D.2* identified two putative candidate genes (TraesCS1D02G292200.1 and TraesCS1D02G292700.1) with a known DNA‐binding domain (Table 3). One of the candidate genes was a plant‐specific B3‐DNA binding protein (TraesCS1D02G292200.1), known to regulate vegetative to flowering transition (Y. Jing et al., 2019).

In conclusion, HNT stress in wheat impacts various agronomic traits including flowering, spike development, stem elongation, and yield‐related traits. QTL mapping using a DH mapping population resulted in the identification of small QTL intervals carrying candidate genes, which should be validated using the functional genomics approaches to understand the underlying molecular mechanisms of night‐time heat stress tolerance. Breeder‐friendly markers should be developed for the QTL intervals to pyramid night‐time heat stress QTLs explaining large phenotypic variance to develop climate‐resilient wheat varieties/germplasm.

## AUTHOR CONTRIBUTIONS

**Kaviraj S. Kahlon**: Conceptualization; data curation; formal analysis; investigation; methodology; resources; software; supervision; validation; visualization; writing—original draft; writing—review and editing. **Kanwardeep S. Rawale**: Data curation; formal analysis; methodology; software; validation; visualization; writing—original draft; writing—review and editing. **Sachin Kumar**: Data curation; formal analysis; writing—review and editing. **Kulvinder S. Gill**: Conceptualization; formal analysis; funding acquisition; investigation; methodology; project administration; resources; supervision; validation; writing—review and editing.

## CONFLICT OF INTEREST STATEMENT

The authors declare no conflicts of interest.

## Supporting information




**Figure S1**. Pearson correlation coefficients between seven agronomic traits under a) control and b) HNT stress.

**Figure S2**. Linear regression analysis performed by considering grain yield (GY) as a dependent variable and PH, DTH, SN, BM, TSW, and TN as independent variables. P value and R^2^ values are given on the graph for each trait. **a—f**: linear regression analysis for traits under control and **g—l**: linear regression analysis for traits under HNT stress.

**Table S1**. Mean performance of flowering and agronomic traits in parent genotypes (KSG1203 and KSG0057) of the DH population under control and high night‐temperature (HNT) stress conditions.

**Table S2**. Interval size in Mb and number of genes in the 25 unique intervals containing 32 QTLs for 7 traits under control, HNT, and HSI.

**Table S3**: Detailed description of domains and motifs identified for QTL intervals shortlisted for candidate gene analysis.

**Table S4**. Top 10 highest yielding DH lines under HNT stress

**Table S5**. Top 10 highest yielding DH lines under control

## Data Availability

Relevant data are included and referenced in this paper and its associated supplementary information.
