# Supplementary material for: Identification and mapping of QTLs and their corresponding candidate genes controlling high night‐time temperature stress tolerance in wheat (Triticum aestivum L.)
Source: Plant Genome. 2024 Sep 24;17(4):e20517. doi: 10.1002/tpg2.20517 (PMC11628910; doi:10.1002/tpg2.20517)
Supplement: Supplementary file 1 [file TPG2-17-e20517-s001.pdf]

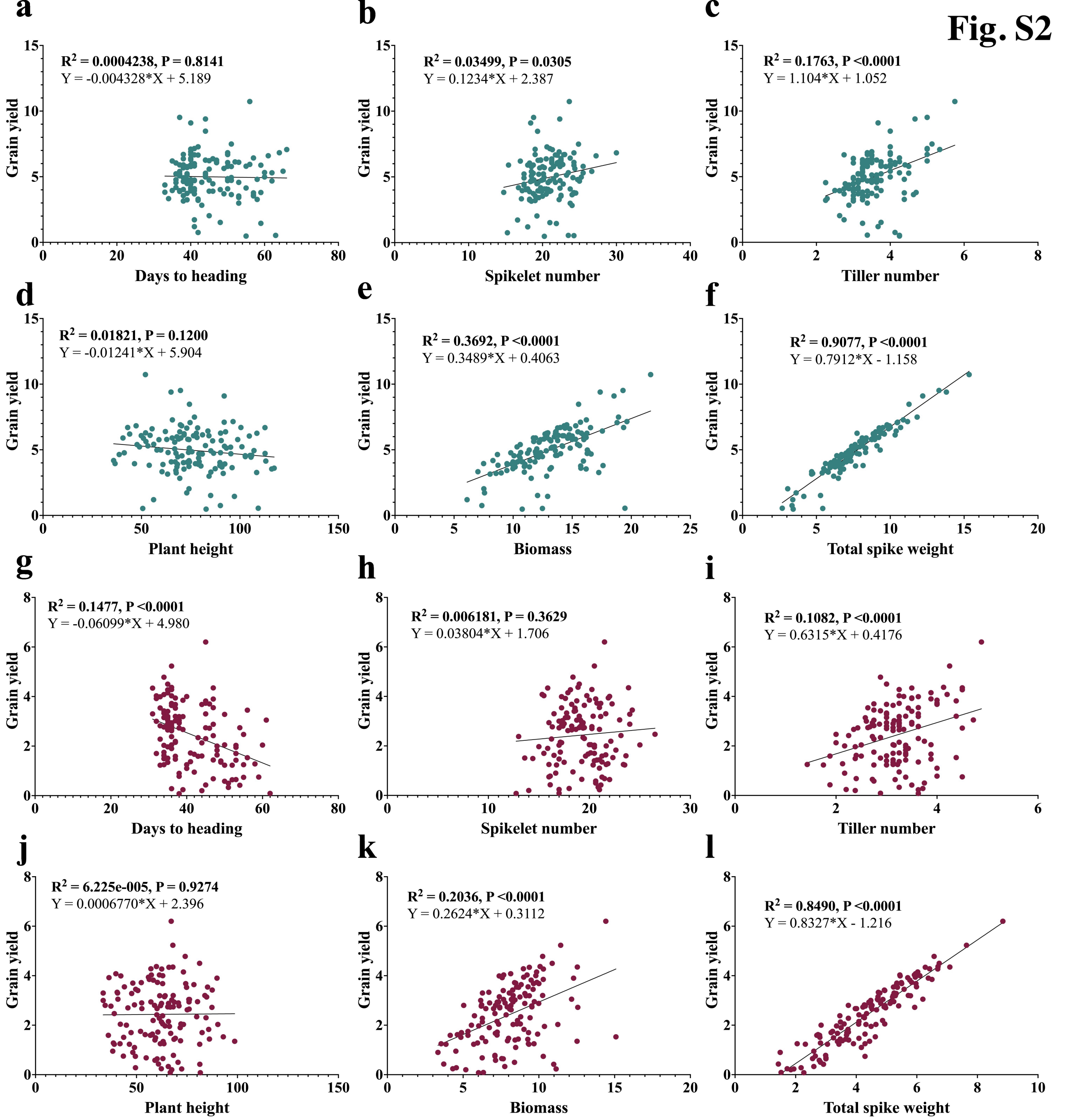

Fig. S2
a
R² = 0.0004238, P = 0.8141
Y = -0.004328*X + 5.189
Days to heading
b
R² = 0.03499, P = 0.0305
Y = 0.1234*X + 2.387
Spikelet number
c
R² = 0.1763, P <0.0001
Y = 1.104*X + 1.052
Tiller number
d
R² = 0.01821, P = 0.1200
Y = -0.01241*X + 5.904
Plant height
e
R² = 0.3692, P <0.0001
Y = 0.3489*X + 0.4063
Biomass
f
R² = 0.9077, P <0.0001
Y = 0.7912*X - 1.158
Total spike weight
g
R² = 0.1477, P <0.0001
Y = -0.06099*X + 4.980
Days to heading
h
R² = 0.006181, P = 0.3629
Y = 0.03804*X + 1.706
Spikelet number
i
R² = 0.1082, P <0.0001
Y = 0.6315*X + 0.4176
Tiller number
j
R² = 6.225e-005, P = 0.9274
Y = 0.0006770*X + 2.396
Plant height
k
R² = 0.2036, P <0.0001
Y = 0.2624*X + 0.3112
Biomass
l
R² = 0.8490, P <0.0001
Y = 0.8327*X - 1.216
Total spike weight
Grain yield
