# Supplementary material for: Identification and mapping of QTLs and their corresponding candidate genes controlling high night‐time temperature stress tolerance in wheat (Triticum aestivum L.)
Source: Plant Genome. 2024 Sep 24;17(4):e20517. doi: 10.1002/tpg2.20517 (PMC11628910; doi:10.1002/tpg2.20517)
Supplement: Supplementary file 3 [file TPG2-17-e20517-s002.docx]

Supplementary Table S3 (Detailed description of domains and motifs identified for QTL intervals shortlisted for candidate gene analysis)
