# Supplementary figures and images for: Identification and mapping of QTLs and their corresponding candidate genes controlling high night‐time temperature stress tolerance in wheat (Triticum aestivum L.)
Source: Plant Genome. 2024 Sep 24;17(4):e20517. doi: 10.1002/tpg2.20517 (PMC11628910; doi:10.1002/tpg2.20517)

**a**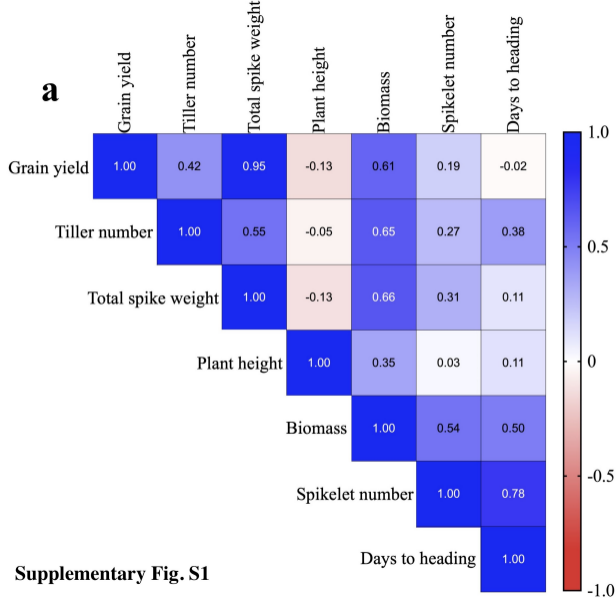**b**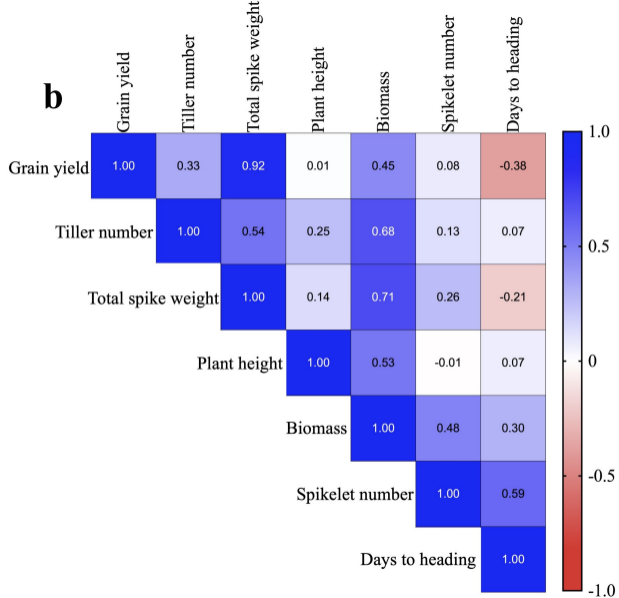

Supplementary Fig. S1

Supplement: Supplementary file 1 — Figure S1. Pearson correlation coefficients between seven agronomic traits under a) control and b) HNT stress. [file TPG2-17-e20517-s006.pdf]

**Fig. S2**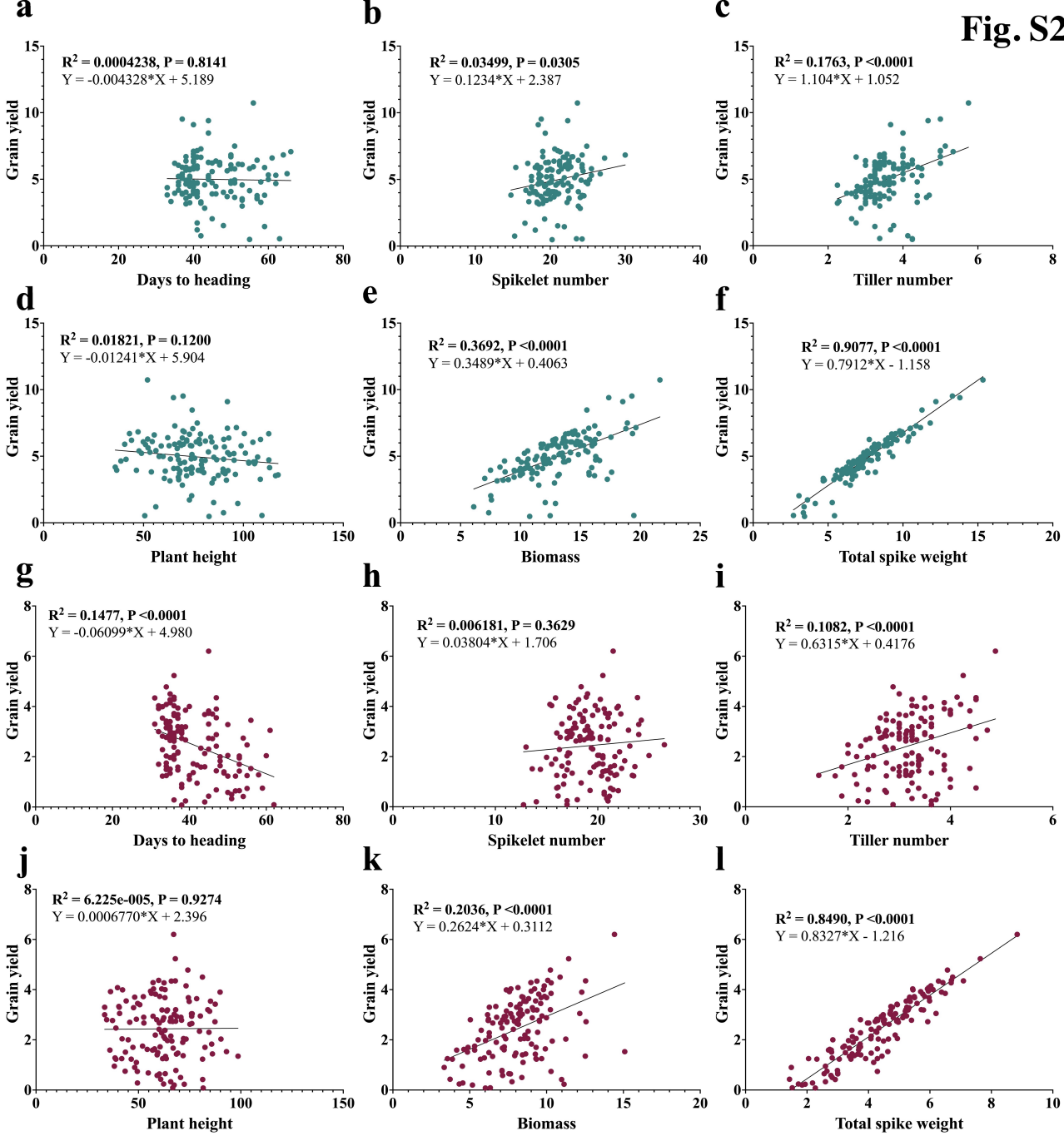

Supplement: Supplementary file 2 — Figure S2. Linear regression analysis performed by considering grain yield (GY) as a dependent variable and PH, DTH, SN, BM, TSW, and TN as independent variables. P value and R2 values are given on the graph for each trait. a—f: linear regression analysis for traits under control and g—l: linear regression analysis for traits under HNT stress. [file TPG2-17-e20517-s001.pdf]
