# Supplementary material for: Identification and mapping of QTLs and their corresponding candidate genes controlling high night‐time temperature stress tolerance in wheat (Triticum aestivum L.)
Source: Plant Genome. 2024 Sep 24;17(4):e20517. doi: 10.1002/tpg2.20517 (PMC11628910; doi:10.1002/tpg2.20517)
Supplement: Supplementary file 3 — Table S1. Mean performance of flowering and agronomic traits in parent genotypes (KSG1203 and KSG0057) of the DH population under control and high night‐temperature (HNT) stress conditions. [file TPG2-17-e20517-s005.docx]

**Supplementary Table S1** Mean performance of flowering and agronomic traits in parent genotypes (KSG1203 and KSG0057) of the DH population under control and high night-temperature (HNT) stress conditions.

| **Traits** | **KSG1203** | | | | | **KSG0057** | | | | |
| --- | --- | --- | --- | --- | --- | --- | --- | --- | --- | --- |
|  | Control mean | HNT mean | RP% | P value | Control mean | | HNT mean | RP% | P value |  |
| DTH | 39.47 | 35.13 | 10.99 | <0.0001 | 51.5 | | 47.33 | 8.09 | 0.0012 |  |
| SN | 18.9 | 17.08 | 9.63 | <0.0001 | 23.94 | | 19.1 | 20.21 | <0.0001 |  |
| PH | 68.15 | 59.67 | 12.44 | 0.001 | 72.41 | | 51.75 | 28.53 | <0.0001 |  |
| TN | 3.1 | 3.08 | 0.55 | 0.9537 | 3.5 | | 2.6 | 25.71 | 0.037 |  |
| BM | 12.14 | 7.98 | 34.31 | 0.0006 | 14.54 | | 5.19 | 64.31 | <0.0001 |  |
| TSW | 8.51 | 5.48 | 35.53 | 0.0008 | 8.76 | | 2.79 | 68.14 | <0.0001 |  |
| GY | 5.95 | 3.87 | 34.94 | 0.0008 | 6.08 | | 1.5 | 75.31 | <0.0001 |  |

DTH: Days to heading, SN: Spikelet number, PH: Plant height (cm), TN: Tiller number, BM: Biomass (gm), TSW: Total spike weight (gm), and GY: Grain yield per plant (gm)

RP%: Relative performance or percent reduction
