# Supplementary material for: Identification and mapping of QTLs and their corresponding candidate genes controlling high night‐time temperature stress tolerance in wheat (Triticum aestivum L.)
Source: Plant Genome. 2024 Sep 24;17(4):e20517. doi: 10.1002/tpg2.20517 (PMC11628910; doi:10.1002/tpg2.20517)
Supplement: Supplementary file 4 — Table S2. Interval size in Mb and number of genes in the 25 unique intervals containing 32 QTLs for 7 traits under control, HNT, and HSI. [file TPG2-17-e20517-s007.docx]

**Supplementary Table S2.** Interval size in Mb and number of genes in the 25 unique intervals containing 32 QTLs for 7 traits under control, HNT, and HSI.

| **QTL Interval no.** | **Chromosome** | **QTLs** | **Interval size (Mb)** | **Number of genes** |
| --- | --- | --- | --- | --- |
| 1 | 5A | *Qph.ksg5A*, *Qph.ksg5A* | 97.48 | 867 |
| 2 | 3B | *Qsw.ksg3B* | 94.61 | 136 |
| 3 | 7B | *Qph.ksg7B* | 68.09 | 534 |
| 4 | 3B | *Qbm.ksg3B* | 67.02 | 298 |
| 5 | 7A | *Qsw.ksg7A* | 62.44 | 688 |
| 6 | 3B | *Qgy.ksg3B* | 43.07 | 379 |
| 7 | 2A | *Qsw.ksg2A* | 40.26 | 57 |
| 8 | 1B | *Qdth.ksg1B* | 23.27 | - |
| 9 | 6B | *Qph.ksg6B* | 19.73 | 286 |
| 10 | 1B | *Qdth.ksg1B* | 19.34 | 174 |
| 11 | 5D | *Qsn.ksg5D* | 19.34 | 251 |
| 12 | 5D | *Qdth.ksg5D*, *Qgy.ksg5D* | 15.79 | 265 |
| 13 | 2A | *Qtn.ksg2A* | 10.9 | 58 |
| 14 | 1B | *Qdth.ksg1B* | 7.28 | 59 |
| 15 | 6A | *Qgy.ksg6A*, *Qsw.ksg6A*, *Qbm.ksg6A* | 6.91 | 63 |
| 16 | 2D | *Qbm.ksg2D*, *Qsw.ksg2D*, *Qsn.ksg2D* | 5.32 | 94 |
| 17 | 1B | ***Qbm.ksg1B*** | 3.55 | **16** |
| 18 | 1D | ***Qsn.ksg1D*** | 3.23 | **25** |
| 19 | 4D | ***Qph.ksg4D*, *Qph.ksg4D*** | 2.54 | **27** |
| 20 | Un | *Qsw.ksgUn* | 2.02 | - |
| 21 | 5D | ***Qbm.ksg5D*** | 1.68 | **15** |
| 22 | 3B | *Qbm.ksg3B* | 1.2 | 21 |
| 23 | 3A | *Qtn.ksg3A* | 1.134 | 16 |
| 24 | 1D | ***Qsn.ksg1D*** | 0.124 | **7** |
| 25 | 5A | ***Qtn.ksg5A*** | 0.0213 | **2** |

The QTLs for control, HNT, and HSI are colored green, red, and blue respectively.

QTLs containing less than 30 genes and identified for night heat stress (bold in table) were selected for candidate gene analysis.
