# Supplementary material for: Identification and mapping of QTLs and their corresponding candidate genes controlling high night‐time temperature stress tolerance in wheat (Triticum aestivum L.)
Source: Plant Genome. 2024 Sep 24;17(4):e20517. doi: 10.1002/tpg2.20517 (PMC11628910; doi:10.1002/tpg2.20517)
Supplement: Supplementary file 6 — Table S4. Top 10 highest yielding DH lines under HNT stress [file TPG2-17-e20517-s003.docx]

**Supplementary Table S4. Top 10 highest yielding DH lines under HNT stress**

| **DH ID** | **DTH** | **SN** | **PH** | **TN** | **BM** | **SW** | **GY** | **%RP (GY)** |
| --- | --- | --- | --- | --- | --- | --- | --- | --- |
| DH52 | 45 | 21.5 | 67.19 | 4.88 | 14.42 | 8.84 | 6.2 | 2.67 |
| DH63 | 36 | 20.5 | 68 | 4.25 | 11.44 | 7.64 | 5.23 | -1.95 |
| DH103 | 34 | 18.38 | 74.13 | 2.88 | 10.25 | 6.57 | 4.78 | 8.08 |
| DH93 | 35 | 19 | 81.25 | 3 | 10.88 | 6.74 | 4.5 | 29.36 |
| DH56 | 36 | 18.88 | 59.88 | 4.13 | 10.08 | 6.7 | 4.37 | 8.77 |
| DH82 | 47 | 23.88 | 68 | 4.5 | 12.54 | 7.09 | 4.35 | 41.93 |
| DH88 | 31 | 15.88 | 66.07 | 3.5 | 9.37 | 6.28 | 4.34 | 0.69 |
| DH150 | 36 | 20.25 | 57.13 | 3.25 | 8.98 | 6.42 | 4.28 | 26.47 |
| DH57 | 35 | 18.13 | 62.5 | 4.5 | 10.3 | 6.69 | 4.27 | 5.95 |
| DH105 | 35 | 17.88 | 73.25 | 3.88 | 9.85 | 6.03 | 4.15 | -16.58 |

%RP (Relative performance) for GY under HNT stress

DTH: Days to heading, SN: Spikelet number, PH: Plant height (cm), TN: Tiller number, BM: Biomass (gm), TSW: Total spike weight (gm), and GY: Grain yield per plant (gm)
