# Supplementary material for: Identification and mapping of QTLs and their corresponding candidate genes controlling high night‐time temperature stress tolerance in wheat (Triticum aestivum L.)
Source: Plant Genome. 2024 Sep 24;17(4):e20517. doi: 10.1002/tpg2.20517 (PMC11628910; doi:10.1002/tpg2.20517)
Supplement: Supplementary file 7 — Table S5. Top 10 highest yielding DH lines under control [file TPG2-17-e20517-s004.docx]

**Supplementary Table S5. Top 10 highest yielding DH lines under control**

| **DH ID** | **DTH** | **SN** | **PH** | **TN** | **BM** | **SW** | **GY** | **%RP (GY)** |
| --- | --- | --- | --- | --- | --- | --- | --- | --- |
| DH151 | 56 | 23.63 | 52 | 5.75 | 21.64 | 15.34 | 10.73 | 66.92 |
| DH165 | 37 | 18.8 | 69.8 | 5 | 19.3 | 13.3 | 9.52 | 86.87 |
| DH171 | 44 | 22.34 | 65 | 4.67 | 17.37 | 13.8 | 9.4 | 88.3 |
| DH178 | 40 | 18.4 | 92 | 3.67 | 18.54 | 12.2 | 9.1 | 56.05 |
| DH184 | 44 | 19.34 | 74.34 | 4 | 15.54 | 11.27 | 8.47 | 68.48 |
| DH82 | 51 | 24.88 | 76.88 | 5.13 | 18.92 | 11.82 | 7.49 | 41.93 |
| DH182 | 42 | 20.6 | 65.7 | 4 | 16.2 | 10.88 | 7.28 | 47.12 |
| DH185 | 41 | 21 | 96.5 | 5 | 19.63 | 11.17 | 7.15 | 58.75 |
| DH189 | 40 | 19 | 92 | 5 | 15.7 | 10.5 | 7.1 | 58.6 |
| DH152 | 66 | 21.34 | 74.67 | 5.34 | 18.8 | 10.47 | 7.07 | 89.4 |

%RP (Relative performance or percent reduction) for GY under HNT

DTH: Days to heading, SN: Spikelet number, PH: Plant height (cm), TN: Tiller number, BM: Biomass (gm), TSW: Total spike weight (gm), and GY: Grain yield per plant (gm)
